# Acquisition process of typing skill using hierarchical materials in the Japanese language

**DOI:** 10.3758/s13414-014-0693-4

**Published:** 2014-05-30

**Authors:** Yuki Ashitaka, Hiroyuki Shimada

**Affiliations:** Graduate School of Maritime Sciences, Kobe University, 5-1-1 Fukae-minami-machi, Higashinada, Kobe, Hyogo 658-0022 Japan

**Keywords:** Acquisition of typing skill, Priming, Japanese language, Typewriting, Fan effect, Chunking, Mora

## Abstract

In the present study, using a new keyboard layout with only eight keys, we conducted typing training for unskilled typists. In this task, Japanese college students received training in typing words consisting of a pair of hiragana characters with four keystrokes, using the alphabetic input method, while keeping the association between the keys and typists’ finger movements; the task was constructed so that chunking was readily available. We manipulated the association between the hiragana characters and alphabet letters (hierarchical materials: overlapped and nonoverlapped mappings). Our alphabet letter materials corresponded to the regular order within each hiragana word (within the four letters, the first and third referred to consonants, and the second and fourth referred to vowels). Only the interkeystroke intervals involved in the initiation of typing vowel letters showed an overlapping effect, which revealed that the effect was markedly large only during the early period of skill development (the effect for the overlapped mapping being larger than that for the nonoverlapped mapping), but that it had diminished by the time of late training. Conversely, the response time and the third interkeystroke interval, which are both involved in the latency of typing a consonant letter, did not reveal an overlapped effect, suggesting that chunking might be useful with hiragana characters rather than hiragana words. These results are discussed in terms of the fan effect and skill acquisition. Furthermore, we discuss whether there is a need for further research on unskilled and skilled Japanese typists.

Typing skills have become ubiquitous worldwide, especially among youths in developed Western countries, because of the popularization of personal computers. Logan and his colleagues (e.g., Crump & Logan, 2010a, 2010b, 2010c; Liu, Crump, & Logan, 2010; Logan & Crump, 2009, 2010, 2011; Snyder, Ashitaka, Shimada, Ulrich, & Logan, 2014; Yamaguchi, Crump, & Logan, 2013; Yamaguchi & Logan, 2014; Yamaguchi, Logan, & Li, 2013) have extensively explored the control of cognitive processes involved in typewriting^1^ and have proposed the two-loop theory of skilled typewriting (see Logan & Crump, 2011, for a summary of this theory; see also Yamaguchi, Crump, & Logan, 2013; Yamaguchi, Logan, & Li, 2013, for an illustration of this theory). The theory proposes that, in skilled typists, the outer loop represents a higher-level control process involved in comprehending sentences, decomposing sentences into words, and submitting the words to the inner loop, whereas the inner loop represents a lower-level control process that is responsible for receiving the words from the outer loop, activating the keystrokes in parallel, and executing them in accurate order.

Although the typing skills in youths in developed Western countries are robust, several researchers have demonstrated that skilled performance can deteriorate by disabling one of several associations that support skilled typing: (a) the association between words and letters (Crump & Logan, 2010b; Logan & Crump, 2011); (b) the association between letters and keys (Liu et al., 2010; Logan, 2003); or (c) the association between keys and finger movements (Crump & Logan, 2010a). Recently, research conducted by Yamaguchi and Logan (2014) demonstrated that a manipulation preventing the skilled typists from chunking in perception, short-term memory, and motor planning could cause previously skilled typists to again be unskilled. The authors suggested that chunking played an important role in the processing of several letters and keystrokes in skilled typewriting. Skilled performance has been thought to develop with chunking, which allows performers to reduce cognitive load in action planning and to concentrate on higher-level action goals (Newell & Rosenbloom, 1981; Yamaguchi & Logan, 2014).

In this study, we used unskilled typists rather than touch typists to examine skill acquisition of the process of typing in the situation in which chunking was readily available. To do so, we used Japanese college students as the participants, because the touch-typing rate in Japanese youths is surprisingly low (approximately 15.4 % in the 18- to 22-year-old demographic; see Director-General for Policy Planning, Cabinet Office, Government of Japan, 2002).^2^ Furthermore, we produced a new keyboard layout consisting of eight keys (and a space key) for each of two groups of typists by removing all of the keys from a standard keyboard, arranging the four keys for four fingers in the left hand and the other four keys for four fingers in the right hand, and adding a space key. This setting ensured new association between the alphabet letter and the typists’ finger movements via the key (see Fig. 1; cf. Crump & Logan, 2010a). To examine the associations between the words and the characters and between the characters and the letters, we explored the process of acquiring typing skills when copying Japanese hiragana words (consisting of a pair of hiragana characters)^3^ using an alphabetic input method through typing on this new keyboard. The alphabetic input method is very popular among Japanese people (approximately 80 %–90 % of college students).^4^

**Fig. 1:**
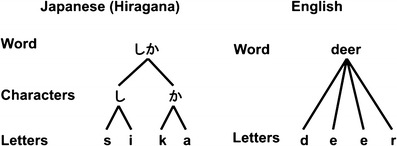
Typing in the Japanese and English languages. The left side shows how a hiragana word is typed using the alphabet input method in Japanese, and the right side shows typing in English. When typing hiragana words in Japanese, the typist must retrieve the alphabet letters associated with the hiragana character. This figure demonstrates that words in the hiragana script in the Japanese language have deeper hierarchical levels than do words in English

Typing Japanese words is more complicated than typing English words. In the Japanese language, the unit of processing is a syllable (especially a mora, which has a constant duration) consisting of either a single vowel (V) or a combination of a consonant and a vowel. Hiragana scripts directly represent these syllables. When copying Japanese words using an alphabetic input method through typing on a keyboard, if all of the words consist of hiragana characters representing syllables, these alphabet letters never appear as visual stimuli.

Generally, Japanese typists must decompose each hiragana character in each hiragana word into a pair of alphabet letters (the first letter denoting a consonant and the second letter denoting a vowel; see Table 1 for the regular syllabic structure of hiragana characters) when typing hiragana words using the alphabet input method in Japanese. In this experiment, words consisting of pairs of hiragana characters were used. Therefore, this task required the typists to type four keystrokes of four alphabet letters for each hiragana word, consisting of a pair of hiragana characters (based on a hierarchical structure in Japanese hiragana words; see Fig. 1 for a comparison of typing in Japanese and English). Each hiragana character consisted of a pair of alphabet letters, allowing us to manipulate the new association (mapping) between the hiragana character and the alphabet letter (see Fig. 2 for the manipulation). Specifically, when typing a hiragana word (e.g., むし; a pair of characters) using the alphabet input method in Japanese, the typists have to type the alphabet letters. For example, in the case of “musi” (four keystrokes), the hiragana characterむis resolved from the pair of alphabet letters m and u; the hiragana character しis decomposed from the pair of alphabet letters s and i.

**Table 1:** Typical pattern of alphabet letters corresponding to hiragana characters

	a	i	u	e	o
	あ (a)	い (i)	う (u)	え (e)	お (o)
k	か (ka)	き (ki)	く (ku)	け (ke)	こ (ko)
s	さ (sa)	し (si)	す (su)	せ (se)	そ (so)
t	た (ta)	ち (ti)	つ (tu)	て (te)	と (to)
n	な (na)	に (ni)	ぬ (nu)	ね (ne)	の (no)
h	は (ha)	ひ (hi)	ふ (hu)	へ (he)	ほ (ho)
m	ま (ma)	み (mi)	む (mu)	め (me)	も (mo)
y	や (ya)		ゆ (yu)		よ (yo)
r	ら (ra)	り (ri)	る (ru)	れ (re)	ろ (ro)
w	わ (wa)				を (wo)

This table indicates the typical pattern of hiragana characters, arranged according to their consonant and vowel components in the Japanese language. The letters in parentheses indicate the letters when typing each hiragana character using the alphabet input method. The first column represents the consonants, and the column headings are the vowels

**Fig. 2:**
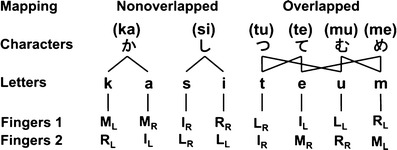
Associations of hiragana characters to alphabet letters: nonoverlapped and overlapped associations. The left side shows the nonoverlapped (unique) mapping between hiragana characters and alphabet letters; the right side shows the overlapped associations between hiragana characters and alphabet letters. Fingers 1 and 2 represent two different key layouts. I_L_ = index finger on the left hand; M_L_ = middle finger on the left hand; R_L_ = ring finger on the left hand; L_L_ = little finger on the left hand; I_R_ = index finger on the right hand; M_R_ = middle finger on the right hand; R_R_ = ring finger on the right hand; L_R_ = little finger on the right hand

We manipulated the new combination of associations (mapping) between the alphabet letter and the hiragana character for each hiragana character using the “overlapped” or “nonoverlapped” mapping. For *overlapped* mapping, the alphabet “*m*”^5^ was used when typing a hiragana character む (*m* *u*), which was included in the hiragana words むし (*m* *u*si) and かむ (ka*m* *u*), and when typing the hiragana character め (*m* *e*), which was included in the hiragana words しめ (si*m* *e*) and めし (*m* *e*si). For *nonoverlapped* mapping, the alphabet “k” was used only when typing a hiragana か (ka), which was included in the hiragana words かて (kate) and かむ (kamu).

In the actual experiment, the alphabet letters were not presented visually; only the hiragana words were presented. The hiragana words in this task had *hierarchical structures* in which each hiragana character at the higher level was composed of a pair of alphabet letters at the lower level. As was described above, the typists had to type the key corresponding to the alphabet letter associated with their finger movement. However, early in the skill development process and in typing training, the typists had to convert each hiragana character used in a hiragana word into a pair of alphabet letters. Since two hiragana characters are associated with a single key location in the overlapped mapping, this situation might produce an overlapping effect between the overlapped and nonoverlapped mappings, as a type of interference (a kind of “fan effect”; see the Discussion section below). Late in the training, chunking should modulate the overlapping effect, which would diminish with the acquisition of typing a series of four keystrokes as a single unit of response, because the association between the key to be pressed and the typists’ finger movements would be strong, and because the key layout in this task had only eight keys (see Yamaguchi & Logan, 2014, for a manipulation in the reverse direction). Furthermore, previous research (Crump & Logan, 2010a) had confirmed that recent experience could influence the degree of skilled typing performance acquired in an individual’s long life history and had considered such an interaction between the recent association and the stored association in long-term memory to be evidence for instance-based skill acquisition (Logan, 1988). Our manipulation (overlapping) of the recent (new) association between the hiragana character and the alphabet letter was anticipated to reveal that the overlapping effect could appear early during typing training and could be diminished by typing the four keystrokes in the hiragana word through chunking later in the training.

Specifically, we expected that the typing speed would be slower in the overlapped than in the nonoverlapped mapping early in skill development. The participants could have started learning associations between individual hiragana characters and key locations early in the training. Since two hiragana characters were associated with a single key location in the overlapped mapping, this would produce interference early in the training. The overlapping effect early in skill development would then decrease during the training, because the unskilled typists would be influenced by the alternative alphabet letter from the hiragana character and would be presented with the new alternative association in the overlapped mapping, whereas the typists late in skill development who had received typing training would have a tendency to type four keystrokes as a single unit through chunking, irrespective of the new association. Conversely, other accounts of the acquisition of skilled performance (Botvinick & Plaut, 2004; Cooper & Shallice, 2000; Lashley, 1951; Norman & Shallice, 1986), such as parallel distributed processing and the schema model, would produce no prediction such as that the overlapping effect would diminish during typing training.

The theory of skilled typing (e.g., Crump & Logan, 2010a, 2010b, 2010c; Liu et al., 2010; Logan & Crump, 2009, 2010; Yamaguchi & Logan, 2014) proposes that reaction time (RT; the interval between word onset and the first keystroke) measures the duration of both outer and inner loops, and that the interkeystroke interval (IKSI; the interval between successive keystrokes) measures the duration of inner-loop processes. By using unskilled Japanese typists, we could closely investigate RT and each IKSI, because each keystroke corresponded to a constant syllable consisting of alphabet letters (the first and third letters represented consonants, and the second and fourth letters represented vowels). This analysis might reveal that chunking is more readily available for a hiragana character (a pair of keystrokes) than for a hiragana word (four keystrokes). If this were the case, the change in the overlapping effect should appear more explicitly in the speed of the second and fourth keystrokes (typing the vowel letters).

## Method

### Participants

Twelve Japanese college students participated in this experiment as part of a course requirement. All were native speakers of Japanese, and all reported normal or corrected-to-normal vision. They reported that they always used the alphabetic-input method when typing Japanese words, and they were non-touch-typists.

### Stimuli and apparatus

The presentation of visual stimuli and the recording of time and accuracy were conducted using the E-Prime software (Version 2.0; Psychology Software Tools, Pittsburgh, PA, USA) for a PC. The visual stimuli included eight Japanese words, each consisting of two hiragana characters (Table 2). The stimuli were presented in white in the center of a 21-in. monitor on a black background. Before testing, the new keyboard was produced by removing all of the keys from a standard keyboard, arranging the four keys for the left hand and the other four keys for the right hand, and adding a space key. The keys all lacked labels, to prevent the typists from looking at the label while typing. We produced two keyboard layouts: One layout consisted of U, M, K, E, S, A, I, and T, corresponding to the keys pressed in the left-to-right order, starting from the left little, left ring, left middle, left index, right index, right middle, right ring, and right little fingers; these keys replaced the A, S, D, F, J, K, L, and “;” keys on a standard keyboard. The other layout consisted of I, K, M, A, T, E, U, and S, corresponding to the keys pressed with the same fingers; these keys also replaced the standard A, S, D, F, J, K, L, and “;” keys.

**Table 2:** Hiragana words used as visual stimuli in the present experiment

Visual word	かて	かむ	しか	しめ	つか	てつ	むし	めし
Typing keys	ka*te*	ka*mu*	sika	si*me*	*tu*ka	*tetu*	*mu*si	*me*si
Meaning	food	bite	deer	deadline	hill	iron	neglect	meal
Familiarity	4.78	5.47	5.75	5.13	4.97	6.06	6.06	5.97
Kanji	糧	噛む	鹿	締め	塚	鉄	無視	飯

The familiarity ratings were based on a 7-point scale, with 7 indicating the highest level of familiarity (Amano & Kondo, 1999). A list of kanji characters was presented once before the experiment, to confirm the meanings of the words, but it was not presented again during the testing. The alphabet letters in italics denote the *overlapped* mapping between the hiragana character and the alphabet letters included in the hiragana word

Consequently, the typists needed to type four keystrokes for a Japanese word consisting of a pair of hiragana characters (e.g., かて, kate). We controlled the familiarity of hiragana words and the frequency of occurrences, in accordance with the work of Amano and Kondo (1999). Half of the keys each (K, S, A, and I) corresponded to the alphabet letters on a one-to-one basis (*nonoverlapped* mapping). The remaining half of the keys (T, M, U, and E) each did not correspond to the specific alphabet letters on a one-to-one basis (*overlapped* mapping; Fig. 2).

For the overlapped mapping, the alphabet letter “*m*,” which was used when typing the hiragana character む (*m* *u*), was included in the hiragana words むし (*m* *u*si) and かむ (ka*m* *u*); it was also used when typing the hiragana character め (*m* *e*) and was included in the hiragana words しめ (si*m* *e*) and めし (*m* *e*si; see Table 2). For the nonoverlapped mapping, the alphabet “k,” which was used only when typing the hiragana か (ka), was included in the hiragana words かて (kate) and かむ (kamu).

### Design and procedure

**Fig. 3:**
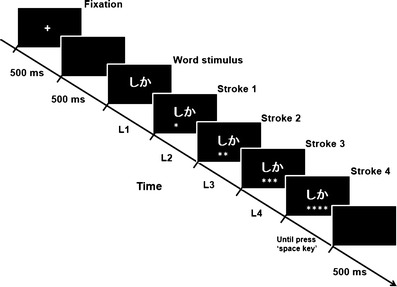
Sequence of events in each trial. L1, L2, L3, and L4 refer to the latencies of the first, second, third, and four keystrokes. L1 and L3 refer to the first and third keystoke latencies, for consonant alphabet keys. L2 and L4 refer to the second and fourth keystoke latencies, for vowel alphabet keys

According to the preceding studies (e.g., Crump & Logan, 2010a, 2010b, 2010c; Liu et al., 2010; Logan & Crump, 2009, 2010; Yamaguchi & Logan, 2014), we separately investigated RTs (L1; the latencies of the first keystroke) and IKSIs (L2, L3, and L4; the latencies of the second, third, and fourth keystrokes^6^; see Fig. 3). Thus, for each response index (L1, L2, L3, and L4), the experiment used an 8 (trial blocks) × 2 (mapping: overlapped or nonoverlapped) within-subjects design. The participants were evenly divided into two groups, each of which used one of the two key layouts. Before testing, the participants received a sheet listing the words in kanji, to clarify the meaning of the hiragana words presented, because the hiragana words could have multiple meanings (Table 2). Furthermore, the participants were informed that hiragana words, rather than kanji words, would be presented one by one on the PC monitor, and they were asked to type the material as quickly and accurately as possible. The eight key labels were spoken verbally to the typists, who were required to memorize them. The participants completed eight blocks of 64 trials individually under normal fluorescent lighting in a soundproof room. Each trial began with a white fixation cross at the center of a black screen for 500 ms, followed by a blank screen for 500 ms, followed by presentation of the hiragana word at the center of the screen (see Fig. 3). The participants were required to press the keys using the alphabetic input method when the hiragana word (consisting of a pair of characters) was presented on the PC display. The participants received feedback in the form of either an asterisk (*), representing a correct keystroke, or an underline (_), representing an incorrect keystroke when typing each key. These symbols were presented approximately 1 cm below a pair of hiragana characters (with each symbol representing the feedback given for typing each alphabet key; see Fig. 3). The hiragana word and the four symbols remained on the screen until the typists pressed the space key. The frequencies of typing the keys corresponding to the eight alphabet letters within each hiragana word were equivalent between the overlapped and nonoverlapped mapping designs. A short break was inserted after each trial block. The participants pressed the space key to begin the next trial block. The experiment lasted approximately 1 h. After testing, the participants were required to recall the placement of the keys.

## Results

Error trials were noted whenever an erroneous keystroke appeared within a word,^7^ in accordance with previous research (Crump & Logan, 2010a). The error analysis revealed a significant learning effect of trial blocks using one-way repeated measures analysis of variance (ANOVA), *F*(7, 77) = 2.73, *MSE* = 33.331, *p* < .05, *η*_p_^2^ = .20; the error percentages were 19.0 %, 13.3 %, 13.0 %, 11.3 %, 10.5 %, 11.1 %, 10.8 %, and 12.4 %, for the first, second, third, fourth, fifth, sixth, seventh, and eighth blocks, respectively. The overall error rate was 12.7 %. Finally, all of the typists recalled the keyboard layout immediately following the 1-h training period.

The following analyses included the correct trials by excluding the error trials. To construct a measure of central tendency, we calculated the median RT and IKSI for each condition in each block for each participant (see Altmann, 2007; Blais & Besner, 2007, for the measurement of medians). We collapsed the data across the two key layouts, because no significant effects emerged between the groups of participants using the two different key layouts (*F* < 1). The RT and IKSI data were separately subjected to a two-way repeated measures ANOVA using trial blocks and mapping as variables. The *p* values for all *F* tests were adjusted using the Greenhouse–Geisser correction for departures from sphericity.

As we described in the introduction, our material of the alphabet letters corresponded to the regular order within each hiragana word (the first and third letters referred to consonants, and the second and fourth letters referred to vowels within the four letters). We performed a two-way ANOVA with repeated measures applied to the trial blocks and the mapping design for each position of the keystroke (viz., the first, second, third, and fourth).

### RT (L1) involved in initiating keystrokes of consonant alphabets

**Fig. 4:**
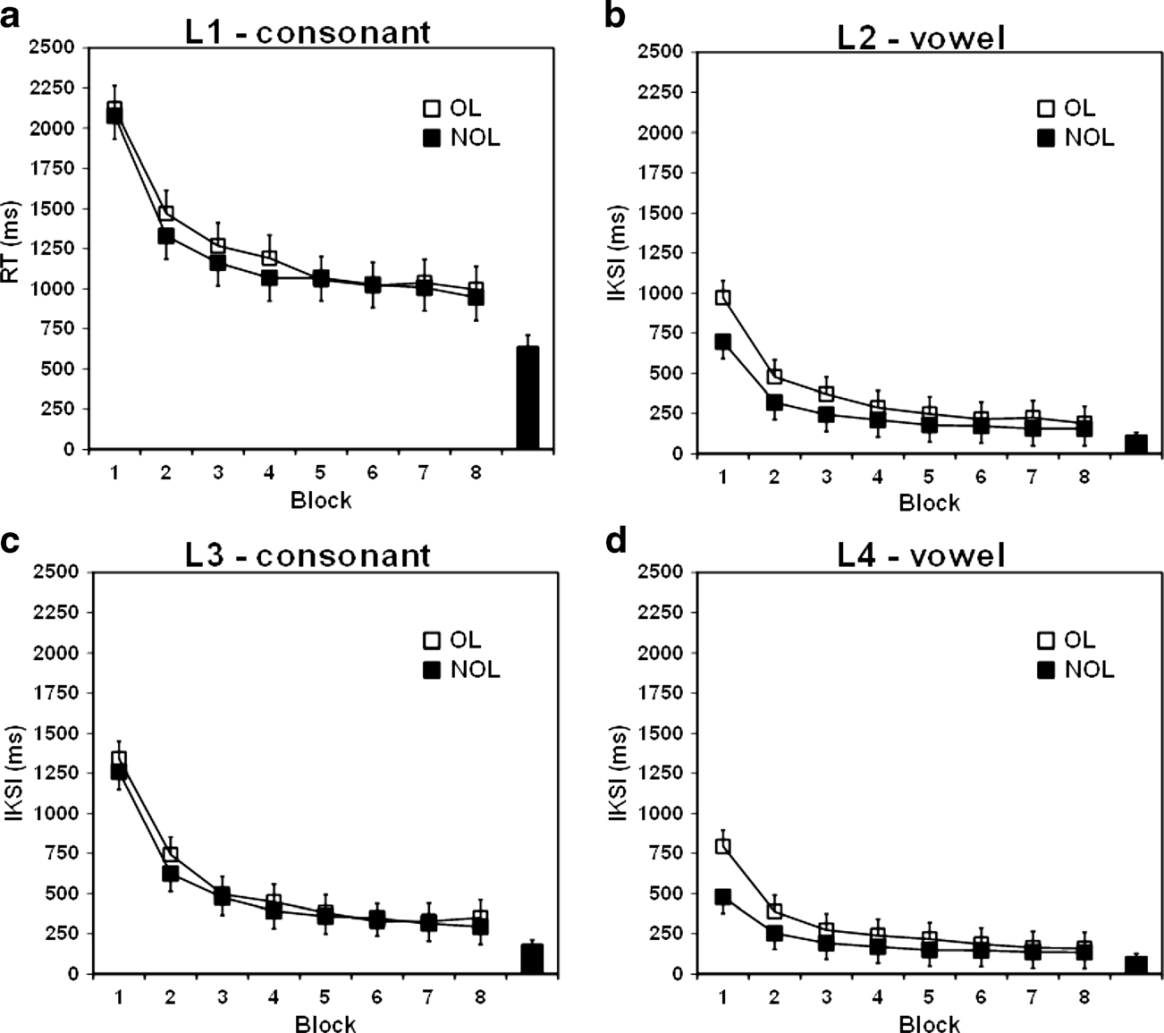
Overlapping effect across blocks during typing training for the RT and each IKSI; (**a**) L1 = RT, the first keystoke latency, for consonant alphabet keys; (**b**) L2 = the second keystroke latency, for vowel alphabet keys—the interkeystroke interval (IKSI) between the first and second keystrokes; (**c**) L3 = the third keystroke latency, for consonant alphabet keys—the IKSI between the second and third keystrokes; (**d**) L4 = the fourth keystroke latency, for vowel alphabet keys—the IKSI between the third and the fourth keystrokes. Error bars represent 95 % within-subjects confidence intervals (Masson & Loftus, 2003). The bar on the right side of each panel represents the median latency in the follow-up experiment, which was conducted using a QWERTY keyboard to type the hiragana words (see the Appendix for the method and results). The same participants participated in both the main and follow-up experiments. NOL = nonoverlapped mapping; OL = overlapped mapping

For the RT (L1) analysis, we found a significant main effect of trial blocks, *F*(7, 77) = 31.9, *MSE* = 104,907, *p* < .001, *η*_p_^2^ = .74, with the earlier stage being marked by slower speeds than the later stage, indicating a learning effect. No significant main effect of the overlapped mapping was apparent, *F*(1, 11) = 2.18, *MSE* = 77,479, *p* = .17, *η*_p_^2^ = .17. We also observed no significant interaction between trial blocks and the overlapping effect, *F* < 1 (Fig. 4a).

### IKSI (L3) involved in initiating keystrokes of consonant letters

The third keystroke was involved in typing consonant letters. The interval between the relevant keystroke and the keystroke before this keystroke (i.e., the interval between the second and third keystrokes; L3) was involved in initiating the keystroke of consonant letters. This interval revealed no overlapping main effect, *F*(1, 11) = 1.34, *MSE* = 66,676, *p* = .26, *η*_p_^2^ = .11, and a significant main effect of trial blocks, *F*(7, 77) = 45.6, *MSE* = 58,599, *p* < .001, *η*_p_^2^ = .81. We found no interaction between overlapping and trial blocks, *F* < 1 (Fig. 4c presents L3). This pattern was similar to the pattern for RTs (L1), which were also involved in typing consonant letters (Fig. 4a).

### IKSIs (L2 and L4) involved in initiating keystrokes of vowel letters

The second and fourth keystrokes were involved in typing vowel letters. The intervals between the relevant keystroke and the keystroke before these keystrokes (the intervals between the first and second keystrokes and between the third and fourth keystrokes; L2 and L4) were involved in initiating the keystrokes of vowel letters. These intervals revealed a significant main effect of overlapping, *F*(1, 11) = 15.1, *MSE* = 35,575, *p* < .01, *η*_p_^2^ = .58; *F*(1, 11) = 17.5, *MSE* = 24,728, *p* < .01, *η*_p_^2^ = .61, for L2 and L4, respectively. At the same time, these intervals revealed a significant main effect of trial blocks, *F*(7, 77) = 19.0, *MSE* = 61,363, *p* < .001, *η*_p_^2^ = .63; *F*(7, 77) = 11.4, *MSE* = 56,273, *p* < .01, *η*_p_^2^ = .51, for L2 and L4, respectively. Furthermore, the intervals revealed a significant interaction, *F*(7, 77) = 4.68, *MSE* = 8,187, *p* < .05, *η*_p_^2^ = .30; *F*(7, 77) = 6.81, *MSE* = 8,121, *p* < .05, *η*_p_^2^ = .38, for the second and fourth keystroke latencies, respectively (Fig. 4b and d present L2 and L4).

## Discussion

In this study, we investigated the skill acquisition process, during which unskilled typists were moving toward becoming skilled typists through typing training when typing Japanese hiragana words on an unfamiliar keyboard. The previous studies of typing skill had always been conducted with skilled typists (thereby leveraging the two-loop theory of the skilled typewriting). The manipulation of our study aimed to improve typing skill through chunking in the direction opposite that used by Yamaguchi and Logan (2014), who degraded skilled into unskilled typists on the learning curve by preventing the skilled typists from chunking. The hierarchical organization of skill has theoretically been thought to be developed through chunking (Newell & Rosenbloom, 1981). We investigated RTs and IKSIs separately regarding the effect of overlapped mapping between hiragana characters and alphabet letters, in the situation in which chunking was readily available. Although, as we described in the introduction, we predicted that typing training would reveal the effect of the overlapped mapping design early in skill training, and that this effect would diminish in late training, this prediction was the case for only L2 and L4, involved in typing vowel letters. However, for RT and L3, both involved in typing consonant letters, this prediction was not correct. These findings suggest that the typists used chunking to make two keystrokes per hiragana character rather than four keystrokes per whole hiragana word.

The effect of the overlapped or nonoverlapped mapping design between the alphabet letters and the hiragana characters, which we found in L2 and L4, might be associated with the fan effect (Anderson, 1974). The fan effect was identified in Anderson’s studies on the actualization of multiple concepts (Anderson, 1974, 1983; Anderson & Reder, 1999), followed by priming studies (Neumann & Deschepper, 1991, 1992), thus identifying the degree of the fan effect as the priming effect’s size (either positive or negative priming). The present experiment revealed that only the latencies of the keystrokes involved in typing vowel letters reflected the fan effect, whereas the latencies of the keystrokes (RT and L3) involved in typing consonant letters did not reflect this effect. As the typists became familiar with the typing materials late in the training, the typists might have learned to process the two keystrokes as a single unit of response to a hiragana character. That would diminish the fan effect, because all two-keystroke sequences would be uniquely associated with a particular hiragana character. Thus, the results could be interpreted as evidence supporting the application of chunking in skilled typewriting (Yamaguchi & Logan, 2014).^8^

However, the alternative possibility is that the reduction observed in the overlapping effect in L2 and L4 might reflect a mere floor effect, in which the typing speed in the nonoverlapped mapping design displayed limited improvement in the last trial blocks (Fig. 4b and d). To investigate the possibility of this interpretation, we conducted a follow-up experiment using a QWERTY keyboard with the same hiragana words and participants as in the present main experiment (see the Appendix for details). Furthermore, we compared the median latencies in the last trial block of the present main experiment with those obtained in the experiment using the QWERTY keyboard by collapsing the overlapped mapping design, because the overlapping effect could not be investigated in the follow-up experiment (see the Method section in the Appendix for further details). Consequently, the typing speeds in the present main experiment for all keystrokes were significantly longer than those in the follow-up experiment. Thus, this finding indicates that the improved typing speed in the last trial block of the present experiment was not limited, and that the typing speed might decrease through further training, suggesting that the diminished overlapping effect could not be attributed to a mere floor effect.

We inferred that we failed to find an overlapping effect for RT and L3 for the following reason. The typing speeds of these keystrokes in the last trial block (970 and 323 ms for RT and L3, respectively) were considerably slower in the present experiment than were those in the follow-up experiment (638 and 183 ms for RT and L3, respectively). Furthermore, the typing speeds for these keystrokes were remarkably slow in the early phase of training (2,099 and 1,300 ms for RT and L3, respectively). The typists might have had to convert a hiragana character into a pair of alphabet letters and look for the key corresponding to the initial consonant for each hiragana character. Thus, the typists might take more time to type the keys of consonant alphabets. The overlapping effect was considerably smaller than the typing speed for these keystrokes; the processing while converting a word into two letters and searching for the correct key required a large amount of time in RT and L3. Thus, the processing involved in the overlapping effect could progress in parallel (rather than additively) with converting and searching, especially early in typing training. The study of unskilled typists has only just begun. Future studies using unskilled typists will need to further investigate the relationship between the skill acquisition process and priming.

By manipulating the direction of learning through setting up a direction opposite to that in Yamaguchi and Logan’s (2014) study, the present experiment confirmed that chunking plays an important role in skill acquisition of typing. We investigated only unskilled typists who were moving toward becoming skilled typists. However, we could not confirm that typists had become skilled late in training. Thus, it appears prudent to exercise caution when comparing the present results to those of previous findings on skilled typists.

To do so, we would need to resolve some problems. First, a method to confirm the accurate assessments of typing speed and accuracy in the Japanese language—such as that observed in Logan and Zbrodoff’s (1998) study in the English language—has never been established. To the best of our knowledge, this study is the first to have investigated Japanese typists from a cognitive psychological perspective. Thus, we need to develop a method through which to assess skill level using hiragana words. Second, it might be difficult to find actual touch-typists in the Japanese population, because we did not find them in this study. As we described in the introduction, according to previous research based only on self-report, the rate of touch-typists is surprisingly low in Japan. Thus, it might be difficult to find touch-typists using a precise assessment method in the Japanese population.

One open question continues to demand further investigation and study. The analysis of the IKSIs revealed that the keystrokes required to type the alphabet letters denoting vowels were significantly shorter than the keystrokes denoting alphabetic consonants over all of the training. This effect was extremely stable. Note that the order of the alphabet letters denoting consonants and vowels within the hiragana character was constant.^9^ The previous studies had indicated that IKSIs were affected by the syllable boundaries within words when typing words in English (Weingarten, Nottbusch, & Will, 2004; Will, Nottbusch, & Weingarten, 2006). It is not clear whether this result involves a Japanese language-specific issue. Future research on Japanese touch typists will resolve this problem.

## Appendix: method and results in the follow-up experiment using a QWERTY keyboard

### Method

#### Participants

The same 12 participants who took part in the main experiment participated in the follow-up experiment using a QWERTY keyboard. The interval between these two experiments was approximately six months.

#### Stimuli and apparatus

The stimuli and apparatus were the same as those that were used in the main experiment, except that we used a regular QWERTY keyboard. In the main experiment, the keys were arranged equally for each finger on the left and right hands for the overlapped and nonoverlapped mapping designs (Table 2). Specifically, the letters were k, a, s, and i for the nonoverlapped mapping condition, and t, e, m, and u for the overlapped mapping condition. Using the QWERTY keyboard, for the nonoverlapped mapping condition, three of the keys to be typed (k, a, and s) were placed on the home row (the same row as the home keys, f and j), indicating easy typing. In contrast, for the overlapped mapping condition, any keys to be typed were not placed on the home row. Thus, the placement of the keys on this keyboard did not allow us to investigate the overlapping effect.

#### Design and procedure

The participants received the only one trial block (64 trials). Thus, the experimental design did not include factors such as the overlapped mapping design and trial blocks, and thus differed from the factors examined in the main experiment. Furthermore, this experiment did not require keyboard recall, again differing from the main experiment.

### Results

We calculated the four keystroke latencies (638, 114, 183, and 108 ms for L1, L2, L3, and L4, respectively; see the black bars on the right sides of the panels in Fig. 4). RT was largest, and the three remaining latencies were short, indicating the use of partial chunking for the four keystrokes; however, L3 was larger than L2 and L4 [*t*s(11) > 6.5, *p*s < .001]. We compared the four keystroke latencies in this experiment with those obtained in the last trial blocks of the main experiment. All of the latencies were significantly shorter than those in the main experiment [*t*(11) = 7.2, *p* < .001; *t*(11) = 3.2, *p* < .01; *t*(11) = 5.5, *p* < .001; and *t*(11) = 2.6, *p* < .05, for L1, L2, L3, and L4, respectively]. The overall error rate was 7.7 %, which was considerably smaller than the error rate (12.7 %) obtained in the main experiment.

## Footnotes

1 There is no explicit differentiation between typing and typewriting (Logan & Crump, 2011). However, “typing” seems to refer to keypresses, as in “typing skill” and “typing training”; “typewriting” seems to involve language production, such as control in typewriting and an expression of language (e.g., Yamaguchi & Logan, 2014). In the present article, we use these terms according this distinction.

2 This survey investigated 3,486 persons, including 823 youths (18- to 22-year-olds), in Japan. Unfortunately, no more recent survey has investigated touch-typing rates in Japan.

3 This article distinguishes hiragana characters from alphabet letters for descriptive purposes.

4 The alphabet input method is recommended in the Guidelines for Elementary Information Education in the Japanese Government (Ministry of Education, Culture, Sports, Science and Technology of Japan, 2010, p. 81). We investigated 88 students (18- to 21-year-olds) enrolled at Kobe University about their preference for using the alphabetic input method in October 2013 for unpublished studies. In all, 87.5 % of the students answered that they consistently used this method. We failed to find other surveys on the preferences of Japanese college students for the alphabet input method.

5 Hereafter, the letters in italics denote the overlapped mapping; the concerned letters are underlined.

6 These latencies refer to the intervals between the relevant keystrokes and the keystrokes before these keystrokes.

7 Error trials were defined on the basis of a word; if any symbols denoting error keystrokes (_) appeared within a word, the PC counted the word as producing an error response.

8 We thank Motonori Yamaguchi for these suggestions regarding the fan effect and chunking.

9 Several studies have investigated the stable and robust syllabic structure of the Japanese language. This syllabic structure has a constant duration of pronunciation as units of processing, which are called *mora* or *morae* (e.g., Hino, Kusunose, Lupker, & Jared, 2013; Hino, Lupker, & Taylor, 2012; Kinoshita, 1998; Tamaoka & Makioka, 2004; Perea, Nakatani, & van Leeuwen, 2011; Sakuma, Sasanuma, Tatsumi, & Masaki, 1998; Verdonschot et al., 2011; Witzel, Qiao, & Foster, 2011).
